# T_mem_ time: Memory T-Cells in Cardiac Allograft Vasculopathy: Editorial to: “Memory T Cells Mediate Cardiac Allograft Vasculopathy and are Inactivated by Anti-OX40L Monoclonal Antibody” by Hao Wang et al.

**DOI:** 10.1007/s10557-013-6507-4

**Published:** 2014-01-03

**Authors:** Michael A. Seidman

**Affiliations:** Centre for Heart Lung Innovation, Department of Pathology & Laboratory Medicine, St. Paul’s Hospital, University of British Columbia, Vancouver, BC Canada

Heart transplantation has been extremely successful as a field, but continues to be plagued by a small number of severe complications. Among these, one of the most frustrating may be cardiac allograft vasculopathy (CAV), a concentric non-atherosclerotic intimal thickening leading to narrowing of the cardiac arterial vasculature and progressive ischemia in allografts [1]. A similar phenomenon is seen in all transplanted organs, and seems to be an immunologically mediated event, despite limited to no response to current immunosuppressive therapies. This CAV phenomenon is one of the most limiting factors in the survival of an individual with a cardiac allograft, and is a common cause of ischemic disease leading to graft failure and retransplantation or death. CAV can involve epicardial coronary arteries, thus clinically mimicking atherosclerotic disease, as well as intramyocardial arteries of any size. Coronary angiography performed on transplant patients is effective at monitoring the epicardial disease, and biopsy features may suggest ischemic damage, but there are no good tools yet for formally monitoring the small vessel narrowing (although surrogate measures of perfusion do exist) [2,3]. Likewise, while stents and bypass are available on a selected basis for the epicardial vessels, no such therapies exist for the small vessel pathology [4–6].

**Fig. 1:**
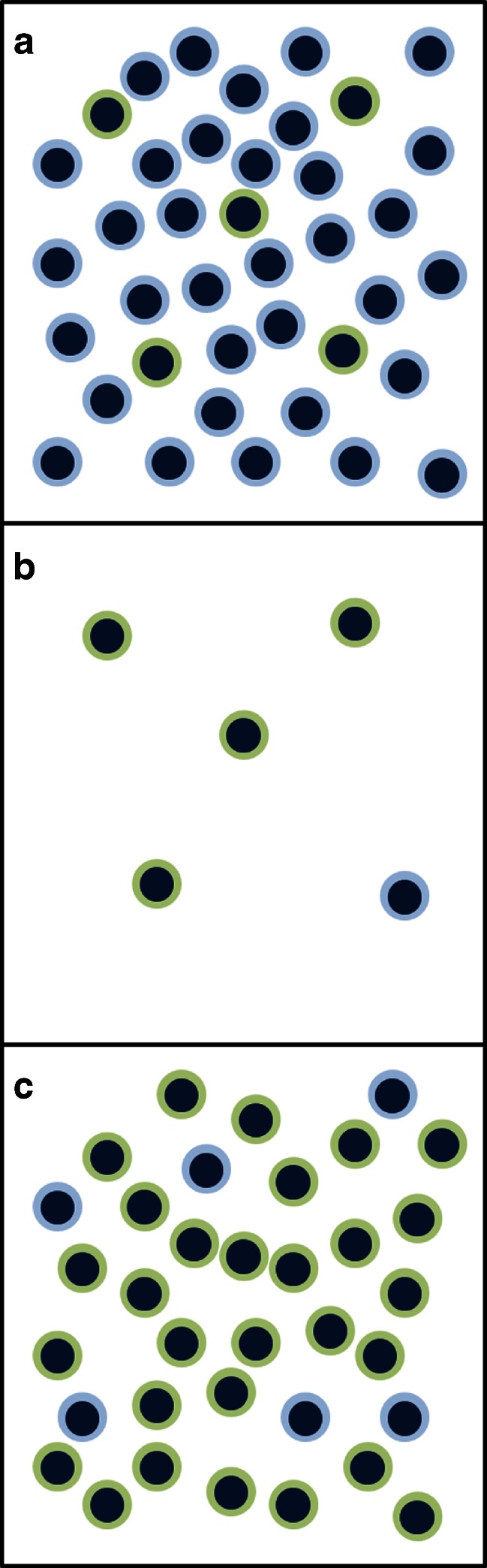
Homeostatic proliferation. **a** In the normal lab mouse or the pre-transplant human, there is a mixed population of T cells in the circulation and tissue, with memory T cells (*green*) representing a relative minority of the population. **b** In immunodeficient mice that have just received adoptive transfer of T cells, or in a transplant patient that has undergone induction of immunosuppression, only a small population of T cells remains, although memory T cells will represent a larger fraction of these cells. **c** Homeostatic proliferation (HP) is the process by which this small number of T cells (panel **b**) proliferates to restore an approximately normal number of T cells in the circulation and tissue; in this situation, memory T cells remain a larger fraction of the overall population

Improved prevention and treatment of CAV will require improved understanding of the pathophysiology of the entity itself. One particularly promising advance in this research is presented in this issue by Wang and colleagues [7]. Using a mouse model, they present compelling evidence that suggests memory T cells may be intimately involved in the development of CAV, and specifically suggest that blockade of the TNF receptor family member OX40 (CD134) may be a therapeutic target in this context. Their model utilizes homeostatic proliferation (Fig. 1), the expansion of lymphocyte populations in a lymphocyte-poor environment [8,9]. Although their model uses lymphocyte deficient mice receiving a T-cell transfer from syngeneic donors, the authors point out the parallels between this situation and the immune reconstitution that occurs in a heart transplant recipient following induction therapies. As laid out by the authors, memory T cells may be much more difficult to eradicate prior to transplantation, and may thus be prone to rapid response on exposure to the allograft. By identifying a candidate tool for targeting these cells, i.e. OX40 blockade, these authors suggest a strategy for potentially stopping CAV before it even begins.

Further, the authors’ work adds to a small number of murine models of CAV, each with slightly different reflections of the potential pathophysiology at work in humans [1]. These models have allowed a number of candidate immunologic therapies for CAV to emerge, including the OX40 blockade suggested in this study. The tools to evaluate human tissues and blood for evidence of analogous mechanisms exist, and thus it is possible to generate the required preliminary data to initiate human investigations. Biologic therapy, i.e. use of monoclonal antibodies and/or recombinant proteins to inhibit biologic pathways in vivo, has become a major tool in oncology, rheumatology, neurology, gastroenterology, and transplantation [10,11]. These highly targeted therapies present powerful opportunities to intervene in processes in patients, but much more study is required. Besides just documenting that the pathways identified in mice are also at work in human, there are a large number of potential complications and side effects that need to be explored, such as excessive immunosuppression leading to opportunistic infection.

Huge advances in immunology continue to inform medical practice, in particular the transplantation field. Ongoing application of that knowledge will lead to improved outcomes, although much work is required.
